# A Potential Magnetic Resonance Imaging Technique Based on Chemical Exchange Saturation Transfer for In Vivo γ-Aminobutyric Acid Imaging

**DOI:** 10.1371/journal.pone.0163765

**Published:** 2016-10-06

**Authors:** Gen Yan, Tao Zhang, Zhuozhi Dai, Meizhi Yi, Yanlong Jia, Tingting Nie, Handi Zhang, Gang Xiao, Renhua Wu

**Affiliations:** 1 Department of Radiology, Affiliated Hospital, Jiangnan University, Wuxi, 214062, China; 2 Department of Medical Imaging, The 2nd Affiliated Hospital, Shantou University Medical College, Shantou, 515041, China; 3 The Mental Health Center, Medical College of Shantou University, Shantou, China; 4 Department of Mathematics and Statistics, Hanshan Normal University, Chaozhou, 521041, China; Henry Ford Health System, UNITED STATES

## Abstract

**Purpose:**

We developed a novel magnetic resonance imaging (MRI) technique based on chemical exchange saturation transfer (CEST) for GABA imaging and investigated the concentration-dependent CEST effect ofGABA in a rat model of brain tumor with blood—brain barrier (BBB) disruption.

**Materials and Methods:**

All MRI studies were performed using a 7.0-T Agilent MRI scanner. Z-spectra for GABA were acquired at 7.0 T, 37°C, and a pH of 7.0 using varying B_1_ amplitudes. CEST images of phantoms with different concentrations of GABA solutions (pH, 7.0) and other metabolites (glutamine, myoinositol, creatinine, and choline) were collected to investigate the concentration-dependent CEST effect of GABA and the potential contribution from other brain metabolites. CEST maps for GABA in rat brains with tumors were collected at baseline and 50 min, 1.5 h, and 2.0 h after the injection of GABA solution.

**Results:**

The CEST effect of GABA was observed at approximately 2.75 parts per million(ppm) downfield from bulk water, and this effect increased with an increase in the B_1_ amplitude and remained steady after the B_1_ amplitude reached 6.0 μT (255 Hz). The CEST effect of GABA was proportional to the GABA concentration in vitro. CEST imaging of GABA in a rat brain with a tumor and compromised BBB showed a gradual increase in the CEST effect after GABA injection.

**Conclusion:**

The findings of this study demonstrate the feasibility and potential of CEST MRI with the optimal B_1_ amplitude, which exhibits excellent spatial and temporal resolutions, to map changes in GABA.

## Introduction

γ-aminobutyric acid (GABA) is an important inhibitory neurotransmitter in the brain that is likely to be involved in almost all signal processing in the central nervous system (CNS) [1]. The concentration of GABA is altered in many brain disorders, including depression, epilepsy, Alzheimer’s disease, and schizophrenia [2–4]. Therefore, a method for in vivo assessment of GABA would be useful for understanding the pathophysiology, diagnosis, and treatment monitoring of related disorders. Magnetic resonance imaging (MRI) is a safe, noninvasive imaging technique that can provide minute structural details on conventional T1/T2-weighted sequences; however, it is not capable of imaging the distribution of neurotransmitters in the brain.

Proton magnetic resonance spectroscopy (^1^HMRS) is one of the techniques used to assess potential disruptions in neuronal integrity and associated neurochemical dysregulation [5, 6]. However, ^1^HMRS requires a long acquisition time and provides poor spatial resolution. GABA resonances measured by ^1^HMRS overlap with those for glutamate (Glu), *N*-acetylaspartic acid (NAA), creatinine (Cr), and macromolecules, thus increasing the challenge for in vivo quantification [7, 8]. Chemical exchange saturation transfer (CEST) is a relatively new MRI technique that can indirectly detect the metabolite content on the basis of exchangeable protons that exchange with bulk water and provides better spatial and temporal resolutions compared with ^1^HMRS [9, 10]. The CEST technique has previously been used to map pH changes based on amide proton (–NH) exchange with bulk water, the protein content in the brain, glycogen changes in the liver, and proteoglycans in the knee cartilage [11–14]. Some small metabolites such as Glu, myoinositol (MI), and Cr were also imaged using CEST in previous studies [15–18].

GABA exhibits a CEST effect between its amine proton (–NH_2_) and bulk water. In the current study, a phantom was used to investigate the concentration-dependent CEST effect of GABA. The extent of contribution from other metabolites was also investigated. Thus, we demonstrated the feasibility and potential of CEST MRI with the optimal B_1_ amplitude, which exhibits excellent spatial and temporal resolution, to map changes in GABA. In addition, we investigated the concentration-dependent CEST effect of GABA in a rat model of brain tumor with blood—brain barrier (BBB) disruption.

## Materials and Methods

### Scanning Device and Acquired Sequences

All MRI studies were performed using a 7.0-T small-animal MRI system (Agilent Technologies, USA) with a 160-mm bore magnet and a standard body coil. In CEST imaging experiments, we utilized an echo planar imaging (EPI) readout sequence with a frequency-selective continuous wave saturation pulse.

### Phantom Imaging

GABA solutions of different concentrations (2, 10, 20, 30, and 50 mM) containing phosphate-buffered saline (PBS) were prepared in small test tubes with the pH adjusted to 7.0. The tubes were immersed in a beaker. CEST Z-spectra for 50 mM GABA were acquired over a ±6.0-parts per million (ppm) range (step size: 0.25 ppm) relative to the bulk water resonance frequency with different B_1_ amplitudes (saturation power) from 0.5 μT to 8.0 μT (step size: 0.5 μT) [19]. The duration of saturation was 3 s, the Band-width of irradiating pulse is 0.333 Hz. The sequence parameters were as follows: flip angle, 90°; repetition time (TR), 4000 ms; echo time (TE), 15 ms; averages, 1; and volume of interest (VOI), 7.0 × 9.0 × 10.0 mm^3^. The total imaging duration was 3 min and 24 s.

In addition, two CEST images from these phantoms were collected at ±2.75 ppm using a 6.0-μT saturation power and a 5-s saturation duration to investigate the concentration-dependent CEST effect of GABA. The sequence parameters were as follows: flip angle, 90°; TR, 6000 ms; TE, 237 ms; slice thickness, 1 mm; average, 32; field of view (FOV), 64 × 64 mm^2^; and matrix, 64 × 64. The total imaging duration was 9 min and 37 s.

In order to evaluate the potential contributions from other major brain metabolites to the CEST effect of GABA, test tubes were filled with solutions containing 50 mM of each metabolite, including Glu, MI, Cr, and choline (Cho), at a pH of 7.0 and immersed in a beaker containing PBS. Two CEST images were also acquired using imaging parameters similar to those described above. All experiments were performed at 37°C.

### Animal Preparation

Four Sprague—Dawley (SD) rats weighing 200–280 g were used to create rat models of brain tumor. All rats were housed in standard facilities and provided ad libitum access to water and rodent food. The animal experiments were performed according to guidelines of the Chinese Animal Welfare Agency and were approved by the Institutional Animal Care and Usage Committee of Shantou University Medical College. We had a protocol in place for the early euthanasia/humane endpoints for animals who became severely ill/moribund during the experiment(s). C6 glioma cells were obtained from American Type Culture Collection (ATCC, USA) and grown in Dulbecco’s modified Eagle medium (DMEM/F12, Gibco, USA) with 10% fetal calf serum and penicillin/streptomycin under a 5% CO_2_ atmosphere at 37°C. Each rat was injected with 1 × 106 exponential-phase C6 glioma cells in the right basal ganglia [20]. To alleviate surgical pain, preemptive analgesia (Buprenorphine 0.05 mg/kg, S.C.) was administered 30 min before operation and post-procedural analgesia (Buprenorphine 0.05 mg/kg, SC q 6–12 hr) was continued for a minimum of 12–24 hours after the animal regained consciousness. After operation, the rats were returned to their home-cage, no cage contained two rats to minimize any enhanced intermale aggression.

### Animal Imaging

Rats were subjected to MRI 2 weeks after the implantation of tumor cells. Anesthesia was induced using 10% chloral hydrate solution and maintained by spontaneous inhalation of a 2%/98% isoflurane/oxygen mixture through a mask with an anesthesia unit. Chloral hydrate has been used as an anesthetic agent widely in animal studies. Compared to the others, it did not significantly alter Heart rate, left ventricular systolic pressure and maximal rate of increase of left ventricular pressure, cell shorting amplitude and survival rate as demonstrated in a recent animal study [21]. The respiration of anesthetized animal was monitored for stability, and the body temperature was maintained at 36–37°C using a water-heated animal blanket. A scout image was initially obtained to verify the position of the subject and the quality of the image. Two CEST images were obtained using the same imaging parameters described above, except that the FOV was 30 × 30 mm^2^ and the slice thickness was 2 mm. After collecting the baseline CEST map, the animals were injected with 2.5 ml of 100 mM GABA solution via the tail vein, following which CEST images were periodically collected for approximately 2.0 h. After finishing MRI scanning, we carried out an euthanasia protocol for all research rats by intraperitoneal injection an overdose of Sodium Pentobarbital (100 or > mg/kg).

### Data Processing

All image processing and data analyses were performed using MATLAB software (version 7.0). Z-spectra were generated by plotting CEST image intensity as a function of the resonance offset of the saturation pulse. CEST asymmetry curves were also generated by plotting the relative water signal difference at frequency offsets from 0 to 6.0 ppm. To correct the B_0_ field inhomogeneity effects, the water shift referencing method [22] was used for B_0_ field mapping, wherein a low-power pulse (B_1_, 0.25 μT) was applied to obtain Z-spectra upon direct water saturation sampled around the water resonance (−1 to +1 ppm). Then, on the basis of the fitted B_0_ inhomogeneity map, all measured Z-spectra curves were also interpolated to 0.01 ppm and shifted accordingly. Finally, the corrected Z-spectra were interpolated back to the 49 sampling points. All analyses were performed on a pixel-by-pixel basis. The CEST signal of GABA was calculated using the equation *(M*_*–2*.*75ppm*_ *− M*_*+2*.*75ppm*_*)/M*_*–2*.*75p*.*p*.*m*_, where *M*_*±2*.*75ppm*_ represents images obtained at ±2.75 ppm from the water resonance [23].

## Results

**Fig 1:**
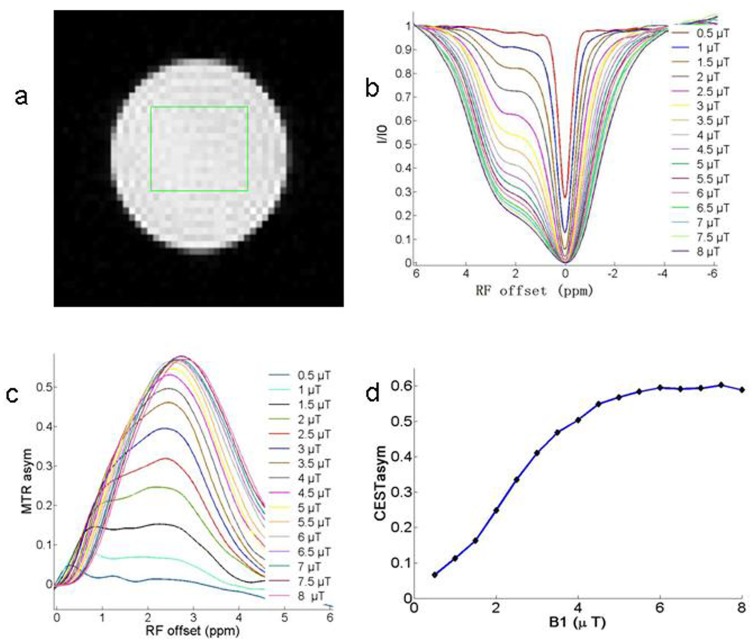
The chemical exchange saturation transfer (CEST) effect of γ-aminobutyric acid (GABA) and its dependence on the B_1_ amplitude. (a) Image of a phantom with 50 mM GABA (pH, 7.0) and the localization voxel indicated. (b, c) Z-spectra and CEST asymmetry curves for 50 mM GABA (pH, 7.0) at different B_1_ amplitudes show the CEST effect at approximately 2.75 ppm downfield to the bulk water resonance. (d) The CEST effect of GABA increases with an increase in the B_1_ amplitude and remains steady after the B_1_ amplitude reaches 6.0 μT.

The CEST peak for 50 mM GABA (pH, 7.0) was clearly observed approximately 2.75 ppm downfield to the bulk water resonance from the Z-spectra at the different B_1_ amplitudes (Fig 1b). The Z-spectral asymmetry plots obtained from the Z-spectra show a broad CEST effect ranging from 1 to 4 ppm from the bulk water resonance (Fig 1c). Fig 1d illustrates that the CEST effect of GABA increased with an increase in the B_1_ amplitude and remained steady after the B_1_ amplitude reached 6.0 μT.

**Fig 2:**
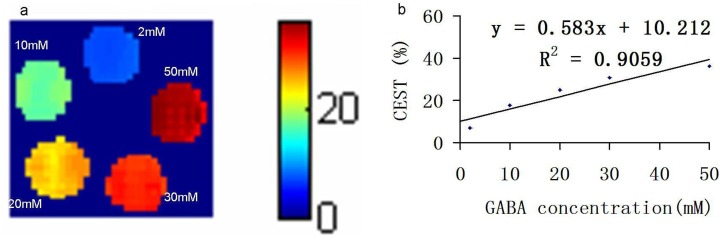
Chemical exchange saturation transfer (CEST) images of a phantom comprising test tubes with different concentrations of γ-aminobutyric acid (GABA) solutions (pH, 7.0) immersed in a beaker containing phosphate-buffered saline (PBS). (a) The region of interest (ROI) shows the CEST contrast color-coded on the original CEST image (2.75 ppm) acquired by the application of a saturation continuous wave with a B_1_ amplitude of 6.0 μT (255 Hz) for 5 s. (b) Linear dependence of the CEST effect of GABA on the GABA concentration, with a slope of 0.58% mM^−^.

By subtraction of two images collected with a saturation frequency selected at ±2.75 ppm, we obtained the CEST images of GABA for a phantom comprising test tubes with different concentrations of GABA solutions (pH, 7.0) immersed in a beaker containing PBS (Fig 2a). Fig 2b shows the CEST contrast dependence on concentrations and demonstrates that the CEST effect of GABA is linearly proportional to the GABA concentration, with a slope of 0.58 mM^−1^, in vitro. This indicates that the CEST effect of GABA can serve as an index of its concentration.

**Fig 3:**
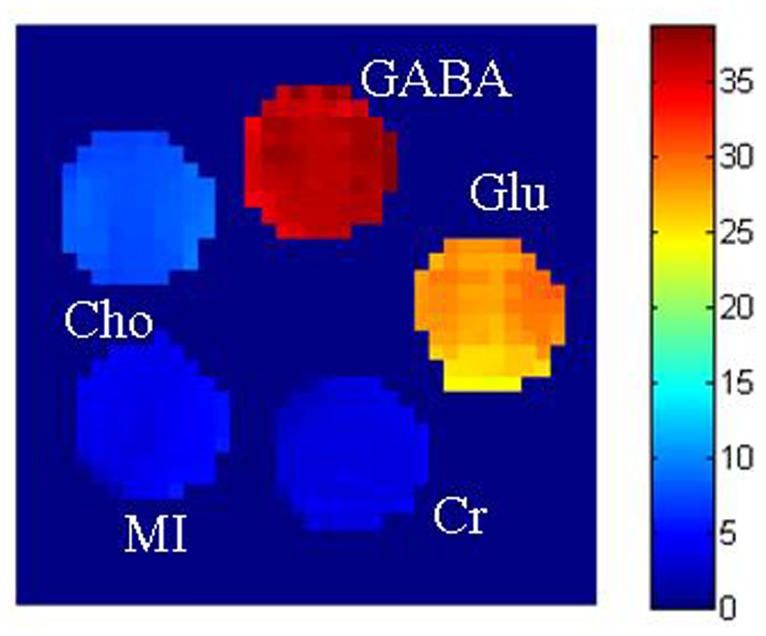
Chemical exchange saturation transfer (CEST) images of a phantom comprising test tubes with solutions containing 50 mM of different metabolites [γ-aminobutyric acid (GABA), glutamine (Gln), myoinositol (MI), creatinine (Cr), and choline (Cho)] at a peak B_1_ amplitude of 6 μT (255 Hz) and a 5-s saturation pulse duration. Except for some contribution from Glu, there was no apparent contribution from the other metabolites at 2.75 ppm.

Fig 3 shows the CEST images of the phantom comprising test tubes with solutions containing 50 mM of different metabolites (GABA, Glu, MI, Cr, and Cho), which were acquired at a peak B_1_ of 6.0 μT and a 5-seconds saturation pulse duration. Except for a small contribution from Glu, the contributions of the other metabolites at 2.75 ppm to the CEST signal of GABA were negligible.

**Fig 4:**
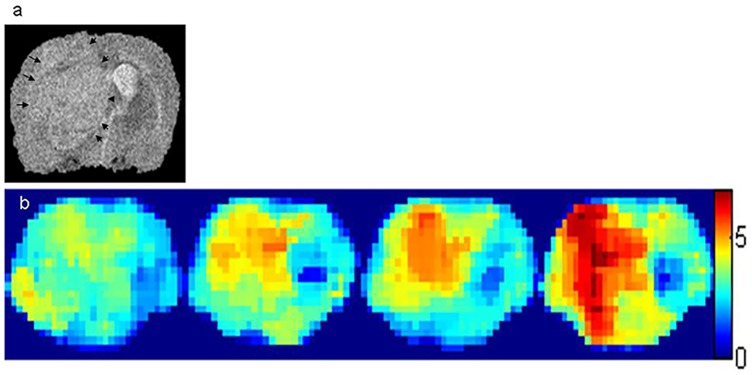
Images of a rat brain with a tumor and disrupted blood—brain barrier (BBB). (a) T2-weighted image demonstrating the tumor and a rectangular region of interest. (b) Chemical exchange saturation transfer (CEST) maps of γ-aminobutyric acid (GABA) in a rat brain with a tumor and a compromised BBB, which were collected at baseline and 50 min, 1.5 h, and 2.0 h after GABA injection, show a gradual increase in the CEST effect of GABA over time.

To investigate the concentration-dependent CEST effect of GABA in vivo, we built a tumor model with a compromised BBB, and CEST maps gathered at baseline and 50 min, 1.5 h, and 2.0 h after intravenous injection of GABA showed a gradual increase in the CEST signal of GABA in the tumor (Fig 4).

## Discussion

The findings of this study demonstrate the feasibility and potential of CEST MRI with the optimal B_1_ amplitude to map changes in GABA. GABA has exchangeable amine protons that exhibit a CEST effect at approximately 2.75 ppm in Z-spectra. Z-spectra reflect the signal intensity of water at a different frequency offset after the application of a saturation pulse, and the exchangeable protons can be selectively irradiated by the application of a radiofrequency (RF) pulse. Their saturated magnetization transfer with bulk water then results in a decreased bulk water signal [24]. Thus, we can indirectly map changes in GABA by imaging the bulk water signal change.

A concentration-dependent CEST signal of GABA was observed in vitro using the imaging parameters described in Methods. An increase of approximately 0.58% in the CEST effect was observed with an increase of 1 mM in GABA. In healthy brains, GABA cannot cross the BBB; however, the compromised BBB in our tumor model allowed the GABA solution to cross over. This is a good way to demonstrate the concentration-dependent CEST signal of GABA in vivo using 7.0-T MRI of rat brains with tumors before and after GABA injection. A gradual increase in the CEST effect was observed after the injection of GABA solution that could cross the compromised BBB, and this was consistent with the results of the phantom studies. Thus, the concentration-dependent CEST effect of GABA observed both in vitro and in vivo provides evidence that the distribution of GABA in the brain can be imaged under some pathological conditions.

The B_1_ amplitude is one of the most important factors affecting the CEST effect of GABA [19]. In the current study, we observed that the CEST effect increased with an increase in the B_1_ amplitude and remained steady after the B_1_ amplitude reached 6.0 μT. An adequate B_1_ amplitude and pulse duration lead to complete saturation; therefore, we selected 6.0 μT as the optimal B_1_ amplitude in our CEST imaging experiments.

We found some contribution from Glu to the CEST effect of GABA under the same experimental conditions, probably because of exchange broadening, which is hard to distinguish in current technology. Therefore, the contamination of Glu will hinder the quantification of CEST effect of GABA in the near future. Contribution from other brain metabolites such as MI, Cr, and Cho was negligible, possibly because of a reasonable difference in the chemical shift [19]. Previous literature reported that glucose had CEST effect at 2.1 and 2.9 ppm, which is very close to 2.75 ppm. With the existence of glucose in vivo, it can be attributed that CEST effect of GABA overlapped with Glucose in some extent [25].

Asymmetric magnetization transfer (MT) effects may confound the CEST signal of GABA because of the contrast magnetization exchange between water molecules bound to larger macromolecules in solid or semisolid phases and free water [26–28]. However, we used a strong saturation pulse (6.0 μT) in this study that minimized MT asymmetry; this was also observed in previous studies [29, 30]. It has also been reported that the concentration of isoflurane used in anesthesia has influence in MTRasym measurements [25]. However, any MT asymmetry effects would result in an underestimation of the actual CEST signal of GABA by competing with the CEST effects of GABA.

In summary, the results of study demonstrate the feasibility of imaging relative changes in GABA using CEST MRI. Although MRS with editing or localized two-dimensional chemical shift methods can also measure GABA levels in vivo or in vitro, particularly at ultrahigh magnetic field strengths, it provides poor resolution. Compared with conventional ^1^H-MRS, the CEST MRI technique used in the present study is noninvasive and nonradioactive and offers high spatial and temporal resolutions for the detection of concentration changes in GABA in the human brain. Further studies should investigate the functions of this technique and explore its potential as a biomarker for the diagnosis and treatment of CNS disorders.

## Supporting Information

S1 Appendix Figure A. CEST Z-spectra of 50 mM GABA with the saturation duration remained 3 s. Figure B. CEST images of a phantom consisting of test tubes with different concentrations of GABA solutions (pH 7.0) immersed in a beaker containing PBS. Figure C. The CEST images of a phantom consisting of test tubes with solution of 50 mM different metabolites (GABA, Glu, MI, Cr and Cho) at peak B_1_ of 6 μT (255 Hz) and 5 seconds saturation pulse duration. Figure D. The T2 weighted image which demonstrates the tumor and a rectangular region of interest. Figure E. Two CEST images of a rat brain with tumor collected at ± 2.75 ppm Figure F. CEST contrast of GABA equaled to *(M*_*–2*.*75ppm*_*−M*_*+2*.*75ppm*_*) /M*_*–2*.*75p*.*p*.*m*_, where *M*_*±2*.*75ppm*_ were images obtained at ± 2.75 ppm from the water resonance respectively.
(PDF)
