# Supplementary material for: A Potential Magnetic Resonance Imaging Technique Based on Chemical Exchange Saturation Transfer for In Vivo γ-Aminobutyric Acid Imaging
Source: PLoS One. 2016 Oct 6;11(10):e0163765. doi: 10.1371/journal.pone.0163765 (PMC5053432; doi:10.1371/journal.pone.0163765)

Supporting Information

S1 Appendix. Figure A. CEST Z-spectra of 50 mM GABA with the saturation duration remained 3 s.

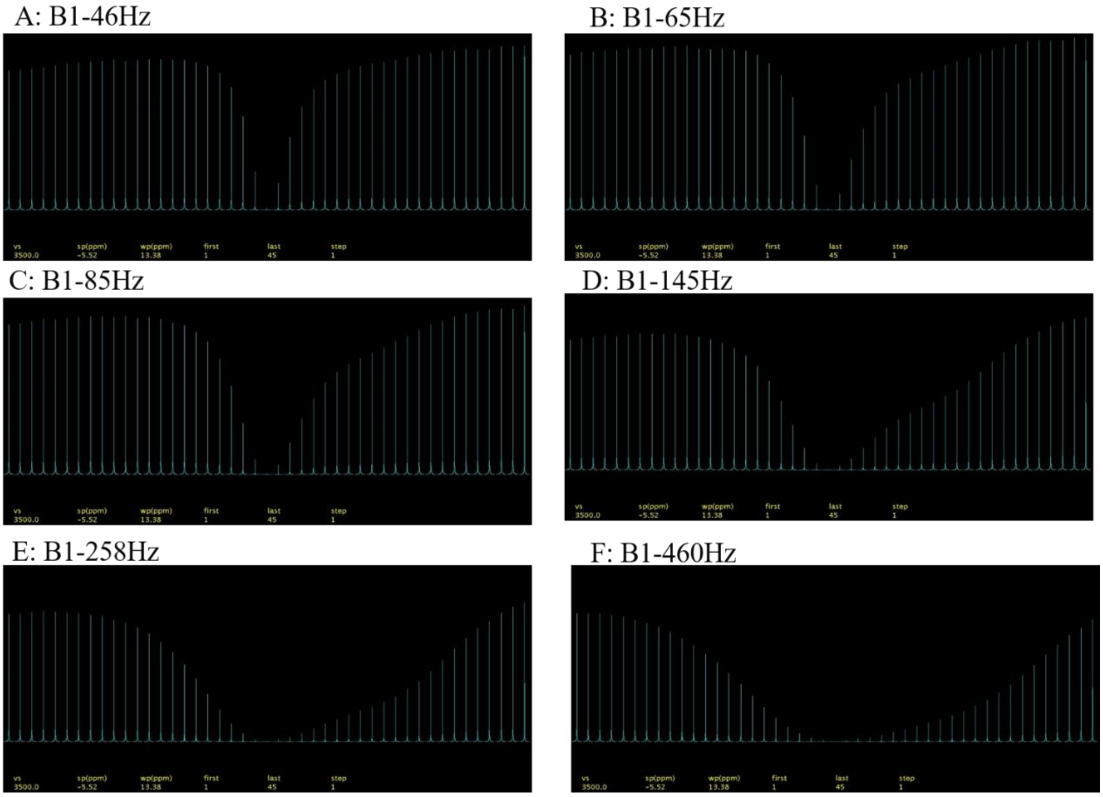

Figure B. CEST images of a phantom consisting of test tubes with different concentrations of GABA solutions (pH 7.0) immersed in a beaker containing PBS.

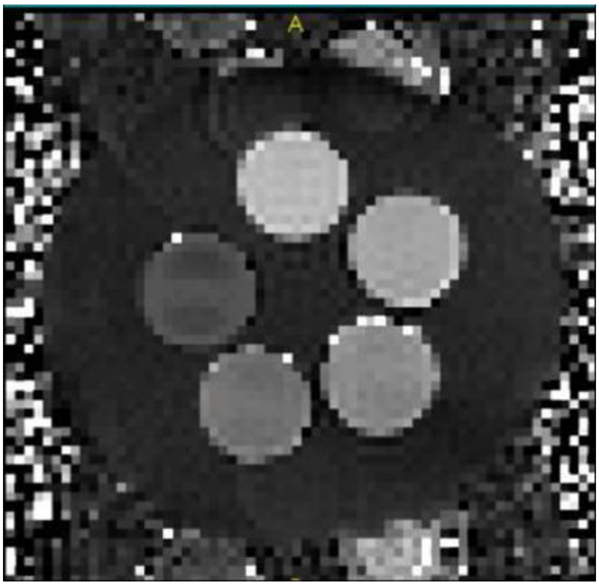

Figure C. The CEST images of a phantom consisting of test tubes with solution of 50 mM different metabolites (GABA, Glu, MI, Cr and Cho) at peak B_1 of 6 μT (255 Hz) and 5 seconds saturation pulse duration.

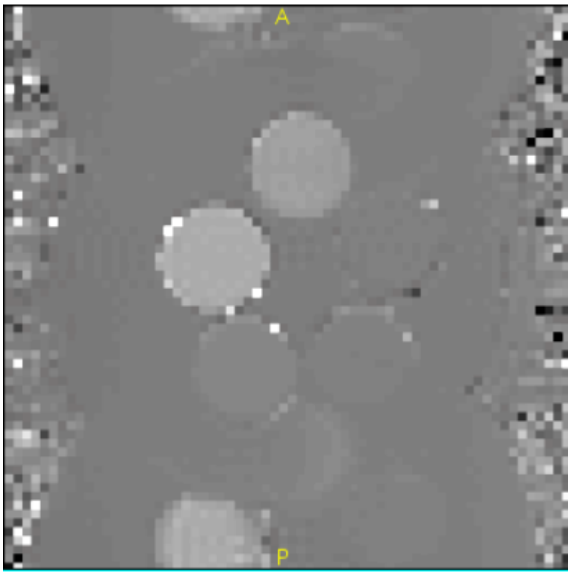

Figure D. The T2 weighted image which demonstrates the tumor and a rectangular region of interest.

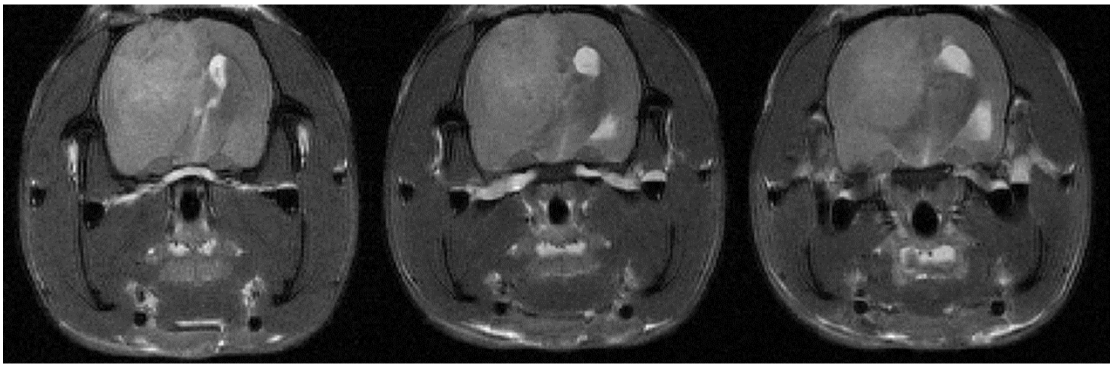

Figure E. Two CEST images of a rat brain with tumor collected at ±2.75 ppm

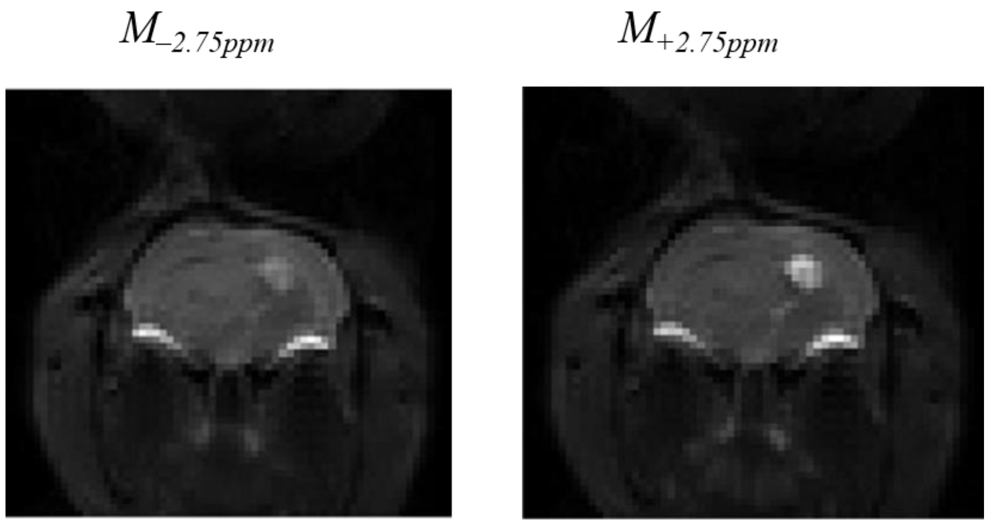

Figure F. CEST contrast of GABA equaled to $(M_{-2.75ppm} - M_{+2.75ppm})$ $/M_{-2.75p.p.m}$, where $M_{\pm 2.75ppm}$ were images obtained at ± 2.75 ppm from the water resonance respectively

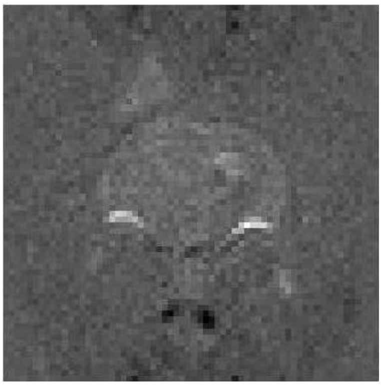

Supplement: S1 Appendix — Figure A. CEST Z-spectra of 50 mM GABA with the saturation duration remained 3 s. Figure B. CEST images of a phantom consisting of test tubes with different concentrations of GABA solutions (pH 7.0) immersed in a beaker containing PBS. Figure C. The CEST images of a phantom consisting of test tubes with solution of 50 mM different metabolites (GABA, Glu, MI, Cr and Cho) at peak B1 of 6 μT (255 Hz) and 5 seconds saturation pulse duration. Figure D. The T2 weighted image which demonstrates the tumor and a rectangular region of interest. Figure E. Two CEST images of a rat brain with tumor collected at ± 2.75 ppm Figure F. CEST contrast of GABA equaled to (M–2.75ppm−M+2.75ppm) /M–2.75p.p.m, where M±2.75ppm were images obtained at ± 2.75 ppm from the water resonance respectively. (PDF) [file pone.0163765.s001.pdf]
